# Rurality and Origin–Destination Trajectories of Medical School Application and Matriculation in the United States

**DOI:** 10.3390/ijgi10060417

**Published:** 2021-06-16

**Authors:** Lan Mu, Yusi Liu, Donglan Zhang, Yong Gao, Michelle Nuss, Janani Rajbhandari-Thapa, Zhuo Chen, José A. Pagán, Yan Li, Gang Li, Heejung Son

**Affiliations:** 1 Department of Geography, University of Georgia, Athens, GA 30602, USA; 2 College of Resource Environment and Tourism, Capital Normal University, Beijing 100048, China;; 3 Department of Health Policy and Management, University of Georgia, Athens, GA 30602, USA;; 4 Institute of Remote Sensing and Geographic Information System, School of Earth and Space Sciences, Peking University, Beijing 100871, China;; 5 August University/University of Georgia Medical Partnership, Athens, GA 30602, USA;; 6 Department of Public Health Policy and Management, School of Global Public Health, New York University, New York, NY 10003, USA;; 7 Department of Population Health Science and Policy, Icahn School of Medicine at Mount Sinai, New York, NY 10029, USA;; 8 Department of Obstetrics, Gynecology, and Reproductive Science, Icahn School of Medicine at Mount Sinai, New York, NY 10029, USA; 9 School of Medicine and Health Management, Tongji Medical College, Huazhong University of Science and Technology, Wuhan 430030, China

**Keywords:** rural physician shortage, medical school application, GIS, origin–destination trajectory, geographic disparity

## Abstract

Physician shortages are more pronounced in rural than in urban areas. The geography of medical school application and matriculation could provide insights into geographic differences in physician availability. Using data from the Association of American Medical Colleges (AAMC), we conducted geospatial analyses, and developed origin–destination (O–D) trajectories and conceptual graphs to understand the root cause of rural physician shortages. Geographic disparities exist at a significant level in medical school applications in the US. The total number of medical school applications increased by 38% from 2001 to 2015, but the number had decreased by 2% in completely rural counties. Most counties with no medical school applicants were in rural areas (88%). Rurality had a significant negative association with the application rate and explained 15.3% of the variation at the county level. The number of medical school applications in a county was disproportional to the population by rurality. Applicants from completely rural counties (2% of the US population) represented less than 1% of the total medical school applications. Our results can inform recruitment strategies for new medical school students, elucidate location decisions of new medical schools, provide recommendations to close the rural–urban gap in medical school applications, and reduce physician shortages in rural areas.

## 1. Introduction

About 15% to 20% of the United States (US) population in rural areas face health inequities, such as worse health care quality and a more severe physician shortage than urban and suburban residents [1]. A recent study commissioned by the Association of American Medical Colleges (AAMC) estimated that the US would face a shortage of between 54,100 and 139,000 physicians by 2033 [2]. The shortage of physicians is particularly acute in rural compared to urban areas. The primary care physician-to-patient ratio in rural areas was only 39.8 physicians per 100,000 persons, compared to 53.3 physicians per 100,000 persons in urban areas [3]. In 2016, only 1.1% of medical students reported a plan to practice in rural or unincorporated areas after completing medical education and training [4]. Unequal geographic distribution of physicians may be worse than the physician shortage itself [5]. The rural physician shortage and the challenge of attracting physicians to practice medicine in rural communities remain a population health challenge.

The federal government established programs such as the National Health Service Corps (NHSC) to address the shortage of rural physicians. The NHSC intended to attract primary care practitioners and other physicians to underserved rural areas [6,7]. Despite these efforts, many NHSC physicians chose not to stay in rural areas once they fulfilled the program commitment [7].

The rural physician shortage in the US has been studied from perspectives ranging from the accessibility to healthcare facilities [8–11], personal choices [12,13] to factors such as neighborhood profiles and the labor market [14–16]. These studies have focused on the existing physician workforce, which is important but could not identify the root causes of the rural physician shortage and geographic disparities. “Rural upbringing” was found as the strongest predictor of choosing a rural practice. Medical school students raised in rural areas were more likely to return to rural settings for practice [7,12,17]. A previous study using data from 399 graduates from a US medical school concluded that geographic origin, birthplace, and college location were related to midcareer practice locations [18]. A study conducted in Japan showed that the regional quota and prefecture scholarship programs encouraged physicians’ rural practice [19].

Our over-arching research question is to analyze and understand the root cause of rural physician shortage. Our study utilizes geospatial origin–destination (O–D) trajectories to assess the patterns of medical school application and matriculation (from hometown to medical school). The objective of the study is to better understand the role of rurality on the geographic variation and disparities in medical school applications and matriculations.

## 2. Materials and Methods

### 2.1. Data and Study Area

We used three datasets in this study: the AAMC data from the American Medical College Application Service (AMCAS), the Student Records System (SRS), and the National Graduate Medical Education (GME) Census in GME Track, a resident database and tracking system for the collection and management of GME data. Our combined dataset included the geographic records of application locations (when students applied for medical schools, also referred to as hometowns in the following text), medical school locations, and GME training sites for all medical school applicants in the US from 2001 to 2015 (N = 581,833 applications, and one applicant may have multiple applications recorded). The AAMC approved our data use agreement. To protect the applicants’ privacy, AAMC aggregated the data spatially to legal county or equivalent level and grouped the data temporally to 5-year intervals from 2001 to 2015. No individual-level data were used in this study. The three datasets, AMCAS, SRS, and GME, were combined by AAMC when they released the data to us. We only worked with the AAMC-processed data, not the original datasets.

Medical school data are publicly available and obtained from the AAMC website [20]. There are currently 155 AAMC member medical schools. However, at the time of data collection and analysis, AAMC members had 149 accredited medical schools in the US and Puerto Rico, and 17 in Canada (not included in the study) at the end of 2015. We geocoded the 149 accredited medical school addresses and applicant counties using the Google Maps Application Programming Interface (API).

Demographic and socioeconomic data at the county level were obtained from the 2010 Decennial Census from the US Census Bureau and the 2010 American Community Survey five-year estimate data, and the National Historical Geographic Information System (NHGIS). Other variables, including medical school counts and the active physician rate, were obtained from the AAMC website and the County Health Rankings & Roadmaps website [21].

Our study included 3220 counties (2015 county delineations) or county statistical equivalents (henceforth counties) located in 50 states and equivalent geographic units (henceforth states) in the US, including Washington, D.C. The AAMC dataset also includes data from some of the 121 county equivalents in US territories (Puerto Rico, US Virgin Islands, Guam, Northern Mariana Islands, and American Samoa). However, due to incomplete data, geographic proximity, and different education systems and policies, we only included 78 units from Puerto Rico in the study. We adopted the rurality classification used by the US Census, which includes four categories based on the percentage of the rural population in a geographic unit [22]. The categories are completely rural (CR) (100% rural population), mostly rural (MR) (greater or equal to 50% but less than 100% rural population), mostly urban (MU) (greater than 0 but less than 50% rural population), and completely urban (CU) (0% rural population). Our medical school application data were from 2001 to 2015, so we used Census 2010 population data. There were 705 (22%) CR, 1187 (37%) MR, 1278 (40%) MU, and 50 (2%) CU counties in our study. The proportions of the population by rurality were 2% (CR), 12% (MR), 79% (MU), and 8% (CU), respectively.

### 2.2. Geovisualization and Spatial Exploration Analysis

We added the Federal Information Processing Standard (FIPS) code for each state and county to the AAMC-SRS data. We then used FIPS as a unique identifier to match the cartographic boundary shapefiles downloaded from the Census. We classified and calculated the application rate (the number of applications per 100,000 persons per year) to mitigate the dominance of some geographic areas with a large population. Furthermore, using different aggregations (annual average, five years, and 15 years) and geographic scales at the county, state, and region levels, we spatially compared the application disparities between different places and time periods, as well as by rurality using ArcMap [23].

### 2.3. Geospatial and Conceptual Trajectories

We utilized geospatial methods to analyze the O–D trajectories of the future and existing physician workforce. A student’s location change, from hometown to medical school and then to GME training location, forms a directed trajectory. We focused on the first segment of the trajectory. Hence, the directed trajectory has two nodes: the application county and the medical school county. A trajectory line represents a student from an application county (origin) to a medical school (destination). From the original 581,833 records of medical school application from 2001 to 2015, after excluding records outside our defined study area and removing missing values, we had 573,031 applications, leading to 259,145 matriculated records and 31,027 O–D trajectories from home counties to medical schools.

The flow mapping method is commonly used to visualize the spatial–temporal trajectory [24–26]. We chose Gephi [27], open-source software with graph and network analysis capabilities, to create flow maps and analyze the trajectory patterns. In our flow maps, the node color symbolizes a state. The origin and destination are connected with a line—with the line width representing the application or matriculation count or rate, and the line color the same as the origin node. We applied both force-based and location-based layouts in Gephi to reveal the potential connection in the trajectories. The force-based layout is generally considered aesthetic and follows a simple principle: linked nodes attract each other, and non-linked nodes are pushed apart. The location-based layout uses longitude and latitude coordinates to set each node’s position in the graph. We selected the Fruchterman–Reingold Algorithm for the force-directed layout (Gephi.org, 2020). The basic idea is to guarantee near nodes (with strong ties) are placed in the same vicinity in the graph, remote nodes (with weak ties) are placed far from each other in the graph, and any overlap is minimized. To synthesize the results, we designed a conceptual diagram to summarize the proportions and trajectories of population, application, and matriculation by rurality.

### 2.4. Statistical Methods: Correlation, Discriminant, and Factor Analyses

We conducted three statistical analyses, correlation, discriminant, and factor analyses, in SPSS [28] and JMP [29] to investigate the relationship between the application rate and rurality. First, we ran a correlation analysis between the application rate and rurality for different time periods. We chose to represent the rurality in two ways: categorical (US census-based four categories) and continuous (percentage of the rural population).

Second, we conducted a discriminant analysis. This analysis allowed data explanation and prediction of disparities by describing group differences and discovering the predictors that discriminate or differentiate between groups [30]. Specifically, seeking to divide the records into two groups, the discriminant analysis focuses on maximizing the “between” sum of squares “SSbetween” (the variation between the two groups) relative to the “within” sum of squares “SSwithin” (the within-group variation). The between-sum of squares is the squared distance between the two group means, and the within-sum of squares is the spread around the means within each group, weighted by the covariance matrix. Intuitively, by maximizing the between-sum of squares and minimizing the within-sum of squares, method discriminant analysis yields the greatest separation between two groups [31]. Since the dependent variable should be categorical in discriminant analysis, we reclassified the application rate into two categories: above average and below average. Then, we used a continuous measure of rurality (% of rural population) to test whether it can distinguish the categorical application rate. The discriminant analysis was conducted using SPSS software [28].

Third, we used a factor analysis to identify the underlying unobservable (or latent) variables, such as underlying socioeconomic factors in a rural region reflected in the observed variables. Using the factor analysis allows us to consolidate many variables, some highly correlated, into just a few independent factors for easy interpretation and mapping. Additionally, the explained variances in factor analysis results indicate the relative importance of different factors and thus differentiate primary and secondary factors [11]. We expected that socioeconomic factors would further explain the geographic and rural disparities of medical school application.

## 3. Results

### 3.1. Spatial Variation of Application and Rurality at the State Level

**Figure 1.**
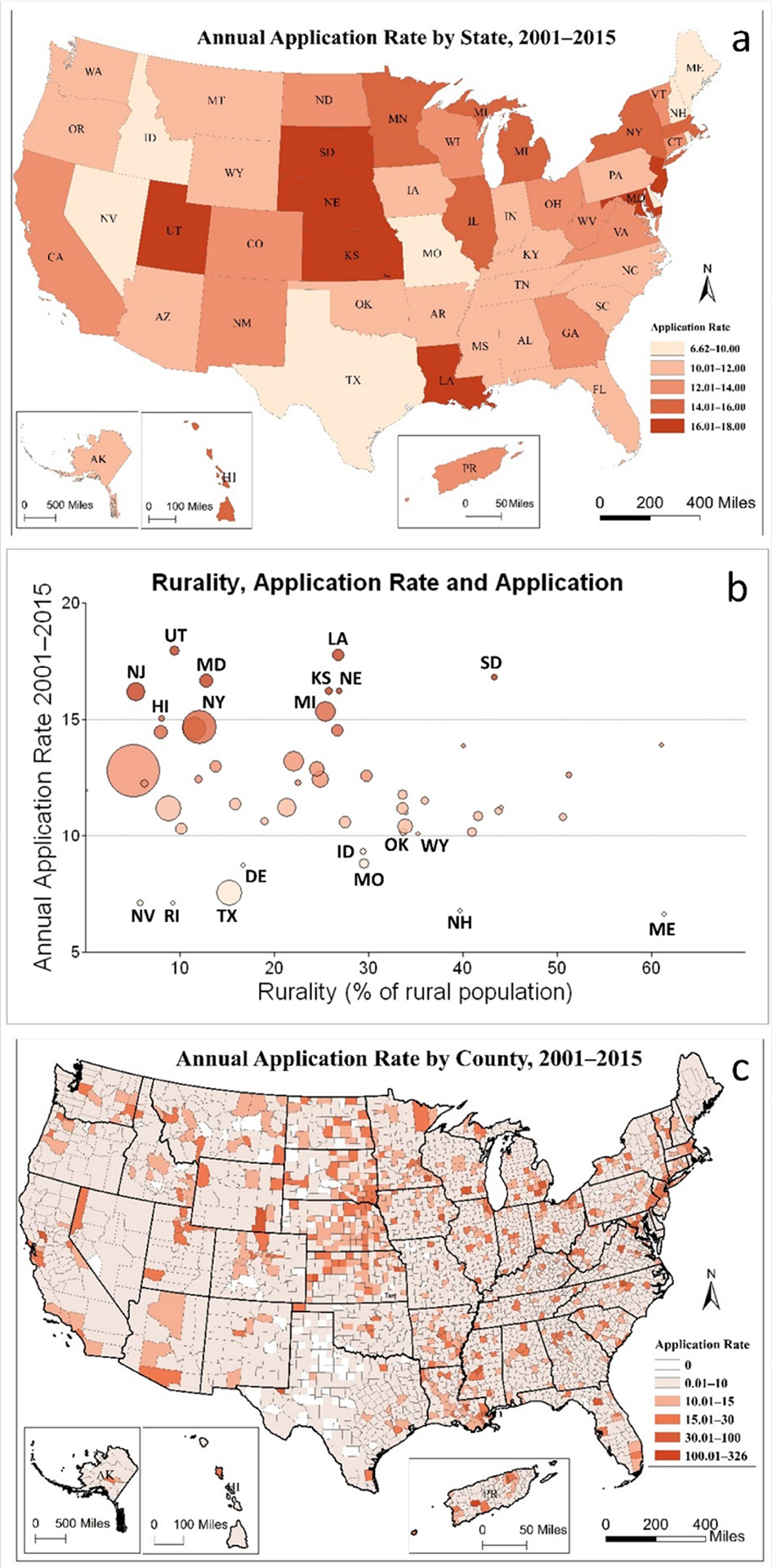
Medical school application rate (applications per 100,000 population per year) and rurality (2001–2015) (**a**) annual application rate by state 2001–2015, (**b**) rurality (x–axis), application rate (y–axis) and application (bubble size), (**c**) annual application rate by county 2001–2015.

Figure 1a illustrates the difference between the medical school application rate at the state-level from 2001 to 2015. The darker color shows the higher application rates, and the lighter color shows the lower rates. Figure 1b presents a scatter bubble plot of the application rate and rurality in the entire 15 years. As shown in the figure, the top ten states with the highest application rates (labeled as bold black at the top of the bubble plot) were Utah (17.96, 9.4%)—meaning the state-level application rate was 17.96 applications per 100,000 persons per year—with 9.42% rurality in the state, Louisiana (17.75, 26.8%), South Dakota (16.81, 43.3%), Maryland (16.64, 12.8%), Kansas (16.22, 25.8%), Nebraska (16.22, 26.9%), New Jersey (16.18, 5.3%), Michigan (15.33, 25.4%), Hawaii (15.03, 8.1%), and New York (14.68, 12.1%). The bottom ten states with the lowest application rates (labeled as bold black at the bottom of Figure 1b) were Oklahoma (10.15, 33.8%), Wyoming (10.09, 35.2%), Idaho (9.3, 29.4%), Missouri (8.79, 29.6%), Delaware (18.70, 16.7%), Texas (7.52, 15.3%), Rhode Island (7.11, 9.3%), Nevada (7.08, 5.8%), New Hampshire (6.76, 39.7%), and Maine (6.62, 61.3%). The top ten states with the highest medical school application rate were mainly in the Midwest and Middle Atlantic regions compared to the bottom ten states mainly in the Northeast, Northwest, and South-Central regions.

Although states with high application rates seem to have low rurality, there is no apparent overall association between these two variables. A simple correlation test confirmed no significant correlation between rurality and the application rate at the state-level. One possible explanation is that the variation of rurality is smoothed out within the state-level, and some information is lost during the data aggregation.

### 3.2. Spatial Variation of Application, Matriculation Rurality at the County Level

The maps of the application rate at the county level include the entire 15-year period (Figure 1c). We also explored the application rate by county at 5-year intervals of 2001–2005, 2006–2010, and 2011–2015. In general, the spatial distribution of high and low values in three time periods at the county-level has no substantial changes visually, and therefore we did not include the maps in the paper. At the county level, higher values of application rate appeared in the middle Atlantic and North-Central regions. Lower values were concentrated in Texas, Oklahoma, and Missouri.

**Figure 2.**
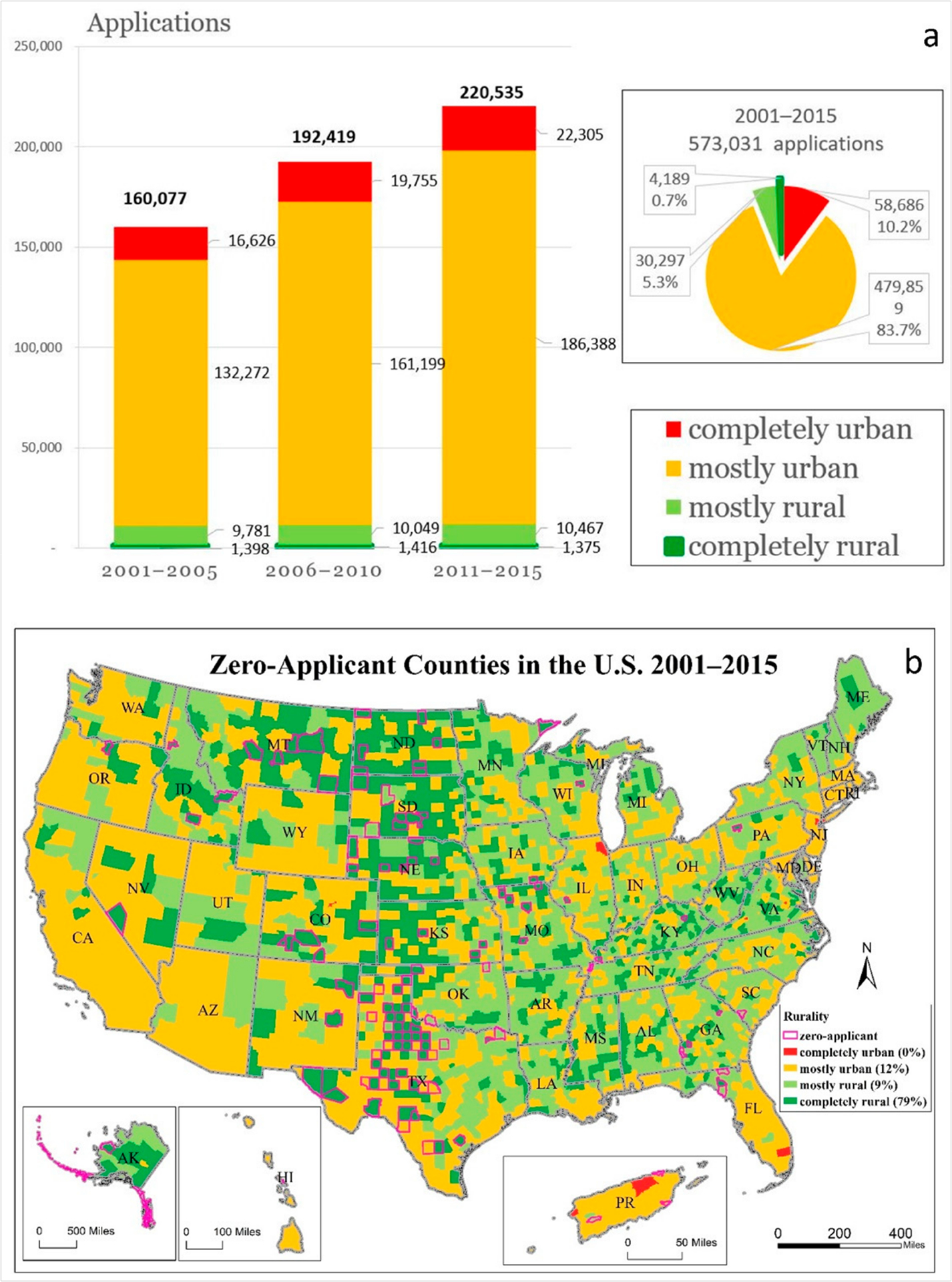
Applications by rurality and zero-application counties (2001–2015), (**a**) total applications by rurality, (**b**) zero-applicant counties in the US.

Figure 2a summarizes the number of application records by rurality at the county level. It shows that over the 15 years and during each of the 5-year periods, although there was an increase in the total number of applications (160,077, 192,419, and 220,535 in the three 5-year periods, 38% increase overall), the applications from CR counties (1398, 1416, and 1375 in the three 5-year periods, 2% decrease) and MR counties (9781, 10,049, and 10,467 in the three 5-year periods, 7% increase) either decreased or had a smaller increase than those of the urban counties (41% increase in MU counties and 34% increase in CU counties).

We mapped the distribution of application rate by rurality and highlighted the 127 zero-applicant counties in 15 years with thick outlines. These counties had the most severe application disparity. (Figure 2b). Among them, 100 were CR (79%), 12 were MR (9%), and 15 were MU (12%) counties. A total of 88% of zero-applicant counties were rural. Geographic disparities do exist in the medical school application. Rural areas have fewer future physicians from the application stage.

Overall, the proportions of the application from CR, MR, MU, and CU counties are 0.7%, 5.3%, 83.7%, and 10.2%, respectively, in the 15-year periods. However, these urban–rural distributions of application are disproportional to the population in those four categories, which are 1.7% (CR), 11.8% (MR), 78.5% (MU), and 8.0% (CU). We used an even distribution as the benchmark, which assumes the same rate of medical school applications for counties of different rurality levels. Compared to the ideal situation of even distribution, the medical school applications were 57% less than it “should be” in CR (i.e., 0.7% is 57% less than the population portion of 1.7%), 55% less in MR (5.3% vs. 11.8%), 7% more in MU (83.7% vs. 78.5%) and 28% more in CU (10.2% vs. 8.0%) counties.

**Figure 3.**
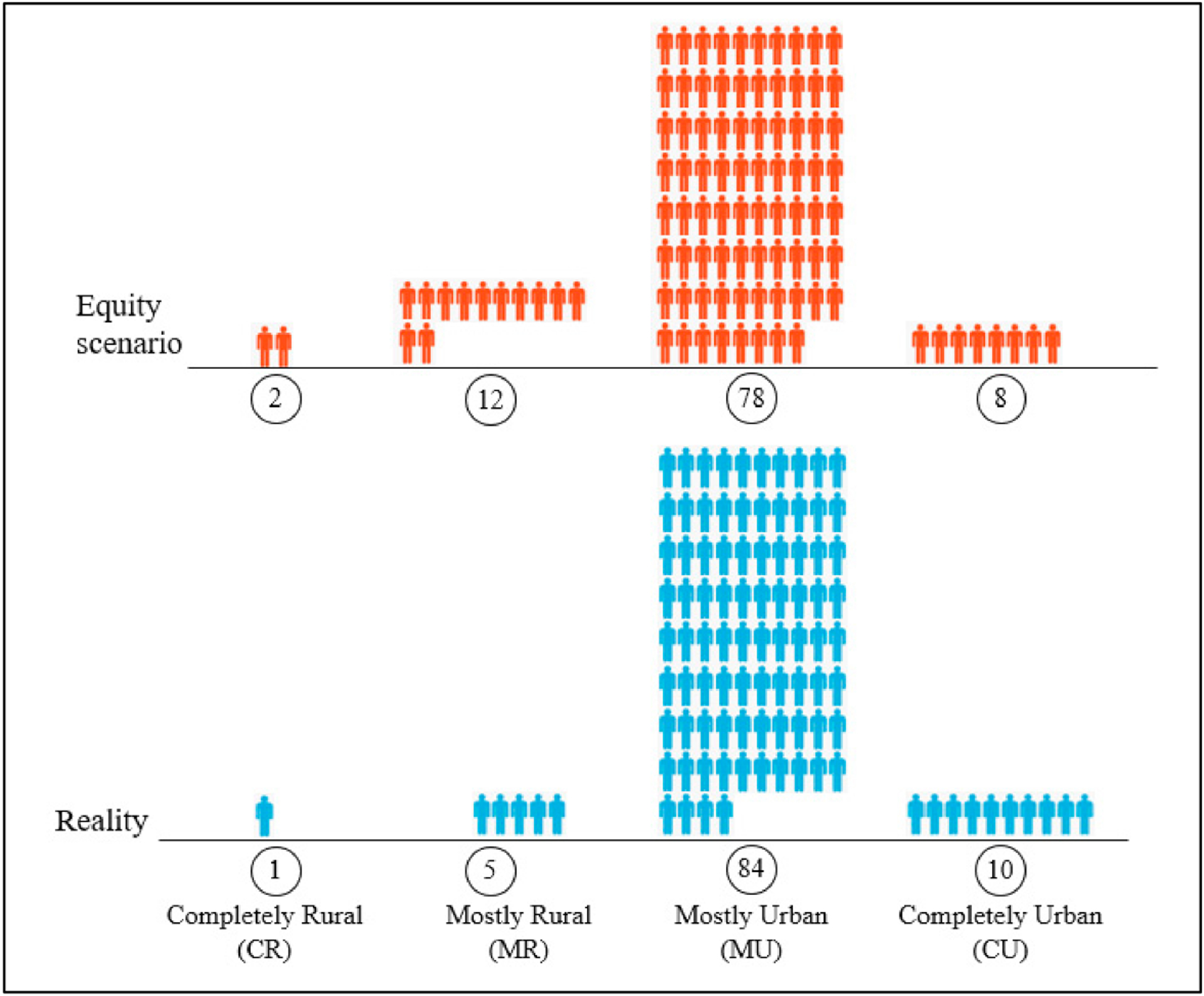
Equity scenario vs. the reality for 100 medical school applicants.

We created an infographic (Figure 3) to demonstrate an equity scenario versus the reality, based on 100 medical school applicants. The equity scenario assumes an equal application rate in all types of counties, so the number of applicants is proportional to the population by rurality (2, 12, 78, and 8 according to the US population by rurality, abovementioned). The reality scenario is based on the proportions of actual applications during the period 2001–2015 (1, 5, 84, 10). Rural areas (CR and MR) have fewer applicants than they would have in an equity scenario.

In our study area, the total number of applications in 15 years was 573,031, and the total number of matriculations was 259,145. The overall mean application rate at the county level was 7.8 applications per 100,000 persons per year, and the mean matriculation rate was 47.6%. However, these measures varied greatly by rurality, as listed in Table 1. Counties with the largest number of applications by rurality were Cook County, IL (for the CU category), Los Angeles County, CA (MU), Geauga County, OH (MR), and Richmond County, VA (CR).

Application rates (per 100,000 persons per year) in rural areas (5.4 in MR, 5.7 in CR) were lower than those in urban areas (13.3 in CU, 9.8 in MU). Except for CU counties, all other types of counties had situations that no one applied to medical school in 15 years. The highest application rates occurred in the Conterminous US in different rurality types of counties, namely: DuPage County, IL (CU), Orange County, NC (MU), Oconee County, GA (MR), and Richmond County, VA (CR). When including non-contiguous states of Alaska and Hawaii, and Puerto Rico, Guaynabo and Adjuntas in Puerto Rico showed the highest application rates for CU and MU areas.

The distribution proportions of matriculations in the four types of counties (10.1% CU, 83.8% MU, 5.3% MR, 0.7 CR) were almost identical to the distribution proportions of application (10.2%, 83.7%, 5.3%, 0.7%), indicating similar matriculation rates by rurality. MU counties provided the largest proportion of matriculated applicants (83.8%). The same four counties with the largest number of applications also provided the largest number of matriculations.

Matriculation rates (% of matriculated applications) among the four rurality types were 44.8% (CU), 45.3% (MU), 45.6% (MR), and 45.5% (CR) for the 15-year average, with the rates in rural counties slightly higher than that in urban counties. The highest matriculation rates occurred in the Conterminous US by rurality: Manassas City, VA (among CU counties), Orange County, NC (MU), Franklin County, IN (MR), and Vilas County, WI (CR). When including non-contiguous states and Puerto Rico, Guaynabo in Puerto Rico showed the highest matriculation rates among CU counties.

Regarding medical schools’ destinations, the dominant O–D types are CU-CU, MU-MU, MR-MU, and CR-MU. The most popular (or largest capacity) medical school destinations were Cook County, IL, for CU applicants (with five medical schools), Philadelphia County, PA, for MU applicants (with four medical schools), Pulaski County, AR, for MR applicants (with one medical school) and Douglas County, NV for CR applicants (with two medical schools).

**Figure 4.**
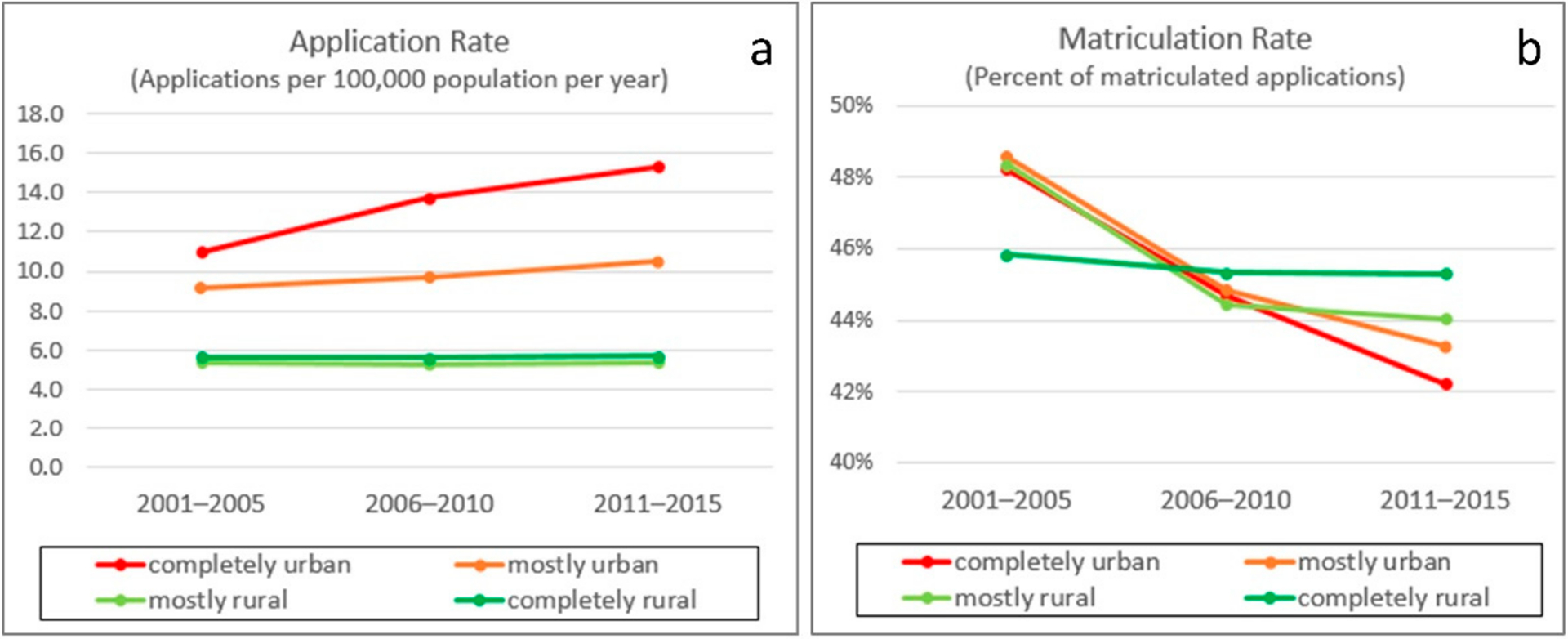
Application rate and matriculation rate. (**a**) Applications per 100,000 population per year, (**b**) the percent of matriculated applications.

Figure 4 presents the application and matriculation rates by rurality across time. The application rates steadily increased in CU and MU counties but stayed almost the same in CR and MR counties. The overall matriculation rate was 45.2% during the 15 years. The matriculation rate showed an L-shaped decrease in 15 years, but the decrease in CR counties was less significant than those in CU, MU and MR counties. As a result, in 2011–2015, applicants from CR counties had the highest matriculation rate (45.3%), followed by MR (44.1%), MU (43.3%), and CU (42.2%), showing an interesting flip from that of 2001–2005.

### 3.3. O–D Trajectories of Medical School Application and Matriculation

**Figure 5.**
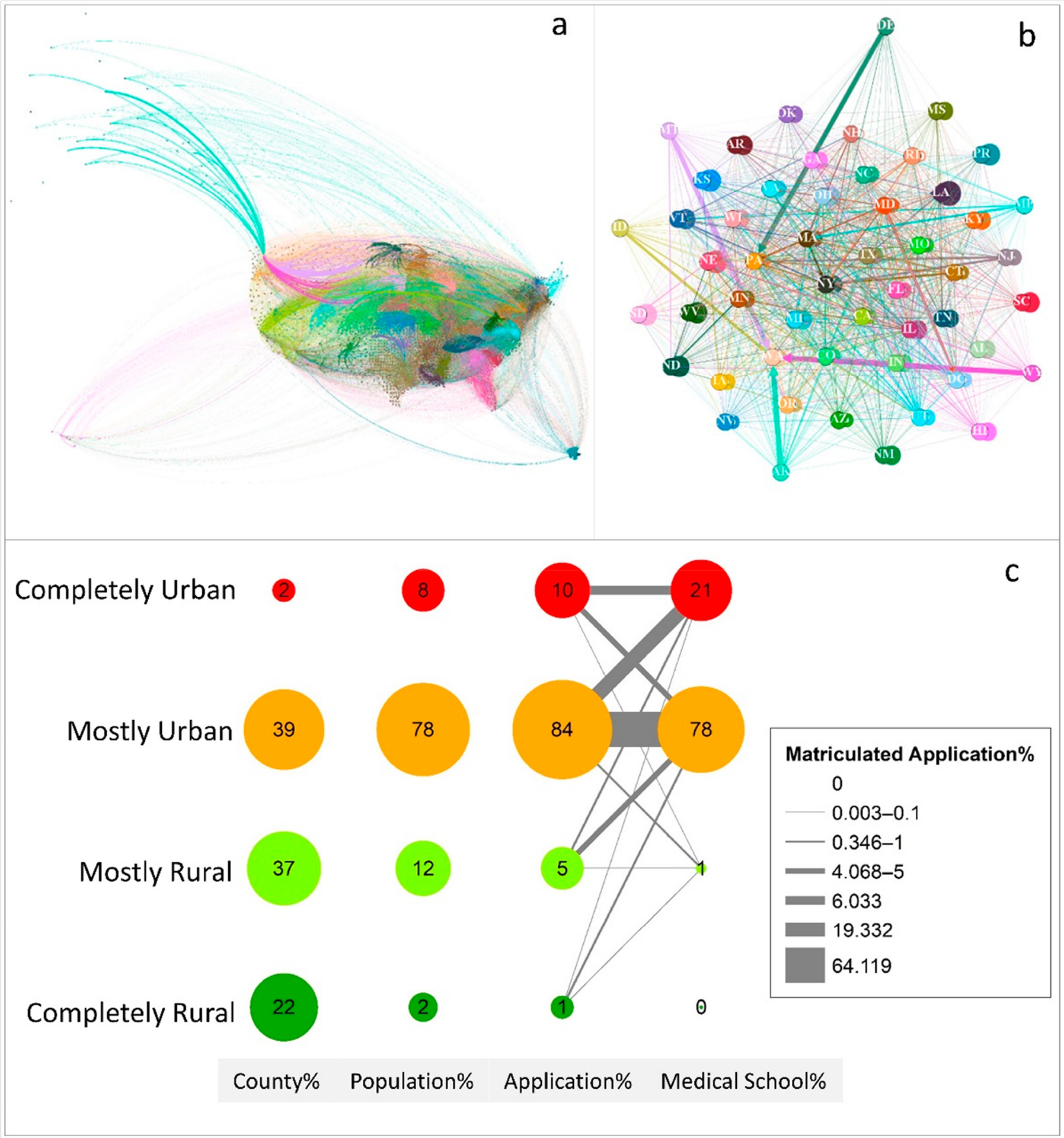
(**a**) County-level application trajectories 2001–2015. (**b**) State-level Fruchterman–Reingold Layout and (**c**) conceptual trajectory.

Figure 5 demonstrates medical schools from application to matriculation O–D trajectories at the county and state levels, as well as a conceptual trajectory. Overall, 62.4% of matriculations in 2001–2015 were in-state. Figure 5a shows the 31,027 county-level trajectories from home counties to medical school counties in 2001–2015 based on 259,145 (out of 573,031) applications with confirmed matriculation. While the raw trajectories preserve the most accurate and detailed data, they are too cluttered to present evident information. In Figure 5b, the Fruchterman–Reingold Layout was applied at the state level to minimize the overlapping and highlight the most connected nodes [32]. The states of California, New York, Texas, Illinois, and Florida were the five largest nodes at center hubs, indicating that they provided the largest numbers of matriculated medical school applications. Some states were on the periphery of the layout, and many of which were the states with small numbers of application, such as Wyoming and Delaware. Delaware was far from the center as it was one of the lowest application states, and it had no medical school in the state. Thus, it had relatively few connections with other states and was away from the center. The widest trajectory from Delaware was to Pennsylvania. When switching the nodes to destination states, the top five destination states were New York, Pennsylvania, Illinois, California, and Texas (graph not shown in the paper).

To further summarize and visualize the information, we generated a conceptual diagram (Figure 5c). In this 16-node diagram, the four rows show categories of the county rurality, and the four columns represent counts of the county, population, application, and medical school. The node size is proportional to the percentage of distribution of each count by rurality, with the total of each column equal to 100. The county and medical school columns depict that, although 59% of the counties are rural (37% + 22%), only 1% of medical schools (one school, to be exact) were located in the MR counties, and 99% were in the urban area. The middle two columns re-emphasize the disproportional relationship between applications and population by rurality. The last two columns summarize the 31,027 county-level O–D trajectories generated from 259,145 medical school matriculations by rurality.

On the concept graph (Figure 5c), the width of the trajectories represents the percent of matriculation during the period 2001–2015. The largest trajectory was from MU counties to MU medical schools (64.12% of all matriculations). Applicants from CR (0% to CR, 0% to MR, 0.66% to MU, 0.08% to CU) and MR (0% to CR, 0.05% to MR, 4.56% to MU, 0.72% to CU) counties were more inclined to apply for medical schools in MU counties. Applicants who came from MU (0% to CR, 0.35% to MR, 64.12% to MU, 19.33% to CU) or CU (0% to CR, 0.04% to MR, 4.07% to MU, 6.03% to CU) counties preferred to apply medical schools that are in the same urbanity type. Medical schools in MR areas were far less attractive to applicants in MU and CU counties, and even applicants in MR counties chose MU or CU over MR.

### 3.4. Statistics Analysis Results

We excluded cases with the potential risk of the small numbers problem [33–37] before running statistical analyses. The correlation analysis at the county level (Table 2) revealed a significant negative association between rurality (% of rural population) and application rate (applications per 100,000 population per year) through the entire 15 years 2001–2015 (with the correlation r = −0.391). Such negative association became stronger in each of the 5-year time periods, 2001–2005 (r = −0.251), 2006–2010 (r = −0.358), and 2011–2015 (r = −0.471). Rurality can explain 6.3% of variance in application rate from 2001–2005 (R^2^ = 0.063), 12.8% in 2006–2010 (R^2^ = 0.128), 17.4% in 2011–2015 (R^2^ = 0.174), and overall 15.3% from 2001–2015 (R^2^ = 0.153). Counties with higher rurality tended to have lower application rates. The tendency has been increasing since 2001.

We divided all counties into two groups, above or equal to the mean application rate and below the mean application rate. We then tested the equality of group means using rurality as the independent variable and found the mean values of the two groups differ significantly (Table 3, *p*-value < 0.001), meaning that rurality can contribute to the discriminant model to group the application rate. The mean value of rurality in the above group was 43.41%, compared to 65.99% in the below group (Table 3). We then ran a discriminant analysis and explored whether the single variable rurality can separate the application rates between above and below groups. We used the continuous variable rurality (i.e., rural population %) to discriminate categorical variable application rate (above or below the mean). The discriminant results showed that rurality alone could significantly separate the medical school application rate into two groups of above mean and below mean. About 68.1% of the counties can be correctly grouped by rurality (shaded cells in Table 4: (745 + 1447)/(1207 + 2010)).

Application is a complex process, and rurality alone could not describe the process. We added 17 socioeconomic variables in seven categories to understand the variations in the application rate further. We used a correlation coefficient matrix to initially screen the correlation coefficients between the socioeconomic variables and the application rate (Table 5). We retained ten variables that were statistically significant and with the absolute value of the correlation coefficient above 0.2 (bolded in Table 5). Due to the multicollinearity among those explanatory variables, we conducted a factor analysis to group them. To better interpret and label different components, we used the varimax rotation technique to maximize the loading of a variable on one factor and minimize the loading on all others [11]. Table 6 presents the rotated factor structure by factor analysis. The ten variables were reduced to three factors and were labeled to reflect major variables captured by each factor: (1) Socioeconomic status, (2) Aging population, and (3) Medical Resource.

Socioeconomic status was the most important factor explaining 39.58% of the total variance. This factor included five variables, and all had positive loading coefficients. The second factor was the aging population, which explained 23.98% of the total variance. The second factor captured three variables and all loading coefficients were positive except for the percent of age between 20 to 34 variables. Medical Resource was the third factor with four variables that explained 11.21% of the total variance. It included the count of medical school, the percent of healthcare practitioners, education and the percent of Asian population.

Finally, we used rurality plus the three factors to rerun the discriminant analysis. We used the variance inflation factor (VIF) test to quantify the severity of multicollinearity. The result showed that multicollinearity was not a significant issue in our factors. The discriminant analysis using both rurality and three factors improved the correctly grouped counties (from 68.1% in Table 4) to 75.5% (Table 7, (633 + 1795)/(1207 + 2010)). There was a 7.4% increase compared with the result of single variable rurality.

## 4. Discussion

### 4.1. Uncertainty of the Medical School Application Data and Analysis

The uncertainties in our data and analysis include those from the aggregation of the medical school applications, the lack of the after-GME career tracks of physicians from the AAMC data, the complex and various understanding and classification of urban and rural areas and populations, and no guarantee of rural practice from rural recruiting.

For example, we adopted the US Census definition of rurality based on the rural population. However, an applicant from an urban county may have gone to high school in a rural community (a rural portion of an urban county), or vice versa. Other scenarios include a rural county near a major metropolitan region that is different from a rural county surrounded by other rural counties. In our future studies, we should look at settings of urban (center county)–urban (surrounding), urban–rural, rural–urban, and rural–rural.

There is also the uncertainty that although rural recruiting could reflect rural practice’s intention, there is no guarantee [38]. In addition to encouraging rural applicants, another worth-trying approach is to build medical schools in rural areas. In Canada, with the concept of “rural pipeline”, and the belief that “physicians have to train or practice in rural or remote areas before they can appreciate the differences between urban and rural medicine”, the first rural, community-based medical school, the Northern Ontario School of Medicine (NOSM), was founded in 2005 [39,40]. NOSM was the first Canadian medical school with the recruiting mandate that the applicants’ demographics reflect the service region’s population. Studies showed that the concept of “rural pipeline” worked, with 25.4% physicians educated in NOSM practicing in rural areas of Ontario compared to 10.3% without NOSM education [41].

### 4.2. The Geography of Medical School Applications and Matriculations

The rural–urban disparities in medical school applications are analyzed geographically and statistically and visualized in various forms such as choropleth maps, infographics, O–D trajectories to conceptual diagrams. Although the total number of medical school applications had gradually increased by 38% from 2001 to 2015, the number of applications increased much less at 7% in MR counties and decreased by 2% in CR counties. The total proportions of applications were 10.2% among CU counties, 83.7% among MU counties, 5.3% among MR counties, and 0.7% among CR counties.

The application rate (per 100,000 persons per year) in rural areas (5.4 in MR, 5.7 in CR) was constantly lower than that in urban areas (13.3 in CU, 9.8 in MU). The number of medical school applications was severely disproportional to the population in the four types of counties by rurality. As a result, CR and MR counties were 57% and 55% less in medical school applications than they should have been in an equity scenario if there were no rural–urban disparities.

There was a considerable amount of geographic variation in the medical school applications and application rates at both the state and county levels. Utah, Louisiana, and South Dakota were the top three states with high application rates, while Maine, New Hampshire, and Nevada were the lowest three. Higher application rates at the county-level appeared in the Middle Atlantic and North-Central regions, and lower-values clustered in Texas, Oklahoma, and Missouri. About 88% of zero application counties in 2001–2015 were in CR or MR counties.

Matriculation rates were 44.8% (CU), 45.3% (MU), 45.6% (MR) and 45.5% (CR) by rurality for the 15-year average. However, the rate decreased in an L-shape pattern and dropped more rapidly in CU, MU, and MR counties. During the last 5-year period, 2011–2015, CR counties had the highest matriculation rate (45.3%), followed by MR, MU, and CU (42.2%). Although CR counties had the highest matriculation rate, their lowest total applications led to very few matriculations, less than 1% of the total—only 1906 in 15 years, or 127 each year from all CR countries in the entire US. At the same time, the total matriculations were 259,145 in 15 years or 17,276 per year.

The O–D matriculation trajectories and the conceptual graph revealed that California, New York, Texas, Illinois, and Florida were the top origin states. The top destination states were New York, Pennsylvania, Illinois, California, and Texas. Applicants from urban counties preferred to matriculate to medical schools in the same urbanity type. Most rural applicants matriculated to MU (86%) counties, with only 13% to CU and less than 1% to MR counties.

Statistical results confirmed the significantly negative association between rurality and application rate throughout 2001–2015. Rurality alone can distinguish application rate to two groups and explain 15.3% of the medical school application rate variation at the county level. Rurality plus three socioeconomic factors can correctly predict 75.5% of the above-mean or below-mean application groups.

Prior research mostly has focused on the existing physician workforce. We argue that through this geospatial analysis and O–D trajectories on both future and existing physician workforce, including medical school application, matriculation, residency, and practice (in future studies) by geographic location, this study provides a better understanding of the flow in medical workforce. Nevertheless, several limitations of this study are worth mentioning. First, we only focused on the beginning part of the trajectory (from the application location to the medical school location), which illustrated the original physician shortage in rural areas. However, tracking the complete trajectory is ideal. Due to the data’s limitation, the trajectories did not go all the way to the practice location. Second, although we might have access to another database, such as the American Medical Association Physician Masterfile, which records the practice location, linking the two databases could introduce additional uncertainties due to the databases’ structures, different variables, various spatial and temporal resolutions, and changes in names and places. Other studies showed that rural variables were strongly associated with rural practice intent, but self-declared rural origins worked better than more “objective” geographic variables [42]. Third, to improve and expand the discussion, we should look into the bigger picture of socio spatial inequalities. We will take all of these into consideration in our future endeavors.

## 5. Conclusions

Tracing back to physicians’ career path origin, we found the medical school application data confirmed that rural areas have significantly fewer applicants in the pipeline to the future workforce. This relationship between rurality and medical school application has important implications for policymakers, regulators, and educators regarding the location allocation and optimization of medical education resources. These outcomes could help new medical schools’ location decisions, among many other factors [43,44]. Rural upbringing or rural pipeline played an essential role in rural practice and rural training during medical schools and/or residencies. The encouraging message from our analysis is that, although the medical school application rate is low in rural counties, the matriculation rate is higher than that in urban areas. This result indicates that once admitted, rural applicants are more likely than their urban peers to proceed with medical education. Although rural recruiting could reflect—but not result in—rural practice [37], it is still one of our most promising and practical strategies to help mitigate physician shortages in the rural areas. Identifying and recruiting medical students from rural areas could be enhanced by building medical school or 4-year regional medical school campuses in rural areas, as in the Canadian’s NOSM establishment case [40]. To summarize, our results inform recruitment strategies for medical schools, elucidate location decisions of new medical schools and regional campuses, and provide recommendations to close the gap in the rural–urban disparities in medical school application to address physician shortages in rural areas.

## Figures and Tables

**Table 1.** County-level application and matriculation counts and rate by rurality from 2001 to 2015.

Rurality (# of Counties)	Measures Count or Rate		Origin County with the Highest Value	Destination (Distribution by Rurality) (Most Popular County)
CU (50)	Applications	(58,686, or 10.2%)	Cook County, IL (127,04)	(**CU**, MU, MR, *CR*) (**6.03%**, 4.07%, 0.04%, *0.00*%) Cook County, IL (4810), CU
	Application rate	**(13.3)**	Guaynabo, PR (40.98) * DuPage County, IL (30.92)	
	Matriculations	(26,272, or 10.1%)	Cook County, IL (5499)	
	Matriculation rate	(*44.8*%)	Guaynabo, PR (62/8%) * Manassas City, VA (53.6%)	
MU (1278)	Applications	(479,859, or **83.7**%)	Los Angeles County, CA (20,800)	(CU, **MU**, MR, *CR*) (19.33%, **64.12**%, 0.35%, *0.00*%) Philadelphia County, PA (10,534), CU
	Application rate	(9.8)	Adjuntas, PR (325.4) * Orange County, NC (73.2)	
	Matriculations	(217,157, or 83.8%)	Los Angeles County, CA (8795)	
	Matriculation rate	(45.3%)	Marion County, IL (74.4%)	
MR (1187)	Applications	(30,279, or 5.3%)	Geauga County, OH (218)	(CU, **MU**, MR, *CR*) (0.72%, **4.56**%, 0.05%, *0.00*%) Pulaski County, AR (543), MU
	Application rate	(5.4)	Oconee County, GA (27.6)	
	Matriculations	(13,810, or 5.3%)	Geauga County, OH (100)	
	Matriculation rate	(**45.6**%)	Franklin County, IN (77.8%)	
CR (705)	Applications	(4189, or *0.7*%)	Richmond County, VA (120)	(CU, **MU**, MR, *CR*) (0.08%, **0.66**%, 0.00%, *0.00%*) Douglas County, NV (16 1), MU
	Application rate	(5.7)	Richmond County, VA (86.5)	
	Matriculations	(1906, or *0.7*%)	Richmond County, VA (42)	
	Matriculation rate	(45.5%)	Vilas County, WI (68.2%)	

• Counties with small numbers problem (<16) were excluded from the rates presented. Application rate is applications per 100,000 persons per year. • Matriculation rate is the percent of matriculated applications. • The highest values both within the study area (including PR) and * conterminous US are presented. • Bold for highest values by rurality, • and italic underline for lowest values by rurality.

**Table 2.** Correlation coefficients between rurality and application rate at the county level.

		Application Rate (2001–2005)	Application Rate (2006–2010)	Application Rate (2011–2015)	Application Rate (2001–2015)
Rurality	r	−0.251**	−0.358**	−0.417**	−0.391**
	Sig.	0.000	0.000	0.000	0.000
	N	3217	3217	3217	3217
	R^2^	0.063	0.128	0.174	0.153

**. Correlation is significant at the 0.01 level (2-tailed).

**Table 3.** Tests of equality of group means.

Application Rate Type		Rurality Mean	Std. Deviation	Wilks’ Lambda	F	df1	df2	Sig.
Counties	Above	43.41	33.08	0.883	424.34	1	3215	0.000
	Below	65.99	28.17					

**Table 4.** Discriminant analysis results with rurality #.

	Application Rate Type		Predicted Group Membership		Total
			Above	Below	
Original	Count	Above	745	462	1207
		Below	563	1447	2010
	%	Above	61.7	38.3	100
		Below	28.0	72.0	100

# 68.1% of original grouped cases correctly classified (shaded).

**Table 5.** Summary statistics of explanatory variables.

Category	Variable	Description	Coefficient
Age	Age 20–34	Percent aged 20–34	**0.320** **
	Median Age	Median age	−0.206**
	62 and Over	Percent of 62 years and over	−0.225**
Gender	Male	Percent of males	−0.185**
Race	White	Percent of White	−0.041*
	Asian	Percent of Asian	**0.363** **
	Black or African	Percent of Black or African American	0.057**
	Hispanic or Latino	Percent of Hispanic or Latino	−0.079**
Socioeconomic Status	Employed	Percent of employed	**0.319** **
	Below Poverty	Percent of population below poverty level	−0.184**
	Median Income	Household median income	**0.338** **
	Mean Income	Household mean income	**0.408** **
Education	Bachelor	Percent of Bachelor’s degree or higher	**0.634** **
Family Environment	Family	Percent of husband–wife families with own children under 18 years	0.086**
Medical Resource	Healthcare Occupation	Percent of healthcare practitioners and technical occupations	**0.399** *
	Active Physician	Active physician rate per 100,000	0.087**
	Medical School	The number of medical schools	**0.329** **

* Correlation is significant at the 0.05 level (2-tailed).

** Correlation is significant at the 0.01 level (2-tailed).

**Bold** variable: significant coefficient >= 0.2.

**Table 6.** Factors with loading coefficients.

_Variables_╲^Factors^	Socioeconomic Status	Aging Population	Medical Resource
Median Income	0.952	-	-
Mean Income	0.932	-	-
Employed	0.821	-	
Bachelor	0.701	-	0.464
Asian	0.442	-	0.417
Median Age	-	0.968	-
62 and Over	-	0.912	-
Age 20 to 34	-	−0.901	-
Medical School	-	-	0.738
Healthcare Occupations	-	-	0.669
% of the total variance explained	39.58	23.75	11.21

**Table 7.** Discriminant analysis results with rurality and three factors ##.

Application Rate Type			Predicted Group Membership		Total
			Above	Below	
Original	Count	Above	633	574	1207
		Below	215	1795	2010
	%	Above	52.4	47.6	100
		Below	10.7	89.3	100

## 75.5% of original grouped cases correctly classified (shaded).

## Data Availability

The data used in this research were acquired through a Data Agreement between the University of Georgia and the AAMC.
